# Videos/Film

**DOI:** 10.1155/1992/69098

**Published:** 1992


### F116

### SIMULTANEOUS COMBINED PANCREAS AND KIDNEY ALLOTRANSPLANTATION IN THE PIG

P.G.SFIKAKIS, P.J.TZARDIS, R.W.G.GRUESSNER, D.E.R. SUTHERLAND
1. Dept. of Surgery, University of Minnesota, Minneapolis Minnesota 55455, U.S.A.
2. Dept. of Surgery, Red Cross Hospital, Athens, Greece

We established a model of en bloc simultaneous pancreas and kidney tranplantation that decreases preservation time, operation time and clamp time. Our method was developed on 32 Yorkshire pigs with the following technique : The donor aorta - with celiac axis, superior mesenteric artery and left renal artery - is anastomosed en bloc to the recipient's aorta in a side-to-oblique fashion. The portal vein is anastomosed end-to-side to the distal vena cava, and the left renal vein end-to-side to the left common iliac vein. The donor duodenum is anastomosed to the bladder to allow monitoring of the urinary amylase for rejection.

The proposed technique facilitates the vascular reconstruction saving one arterial anastomosis and additionally saves about 50min of operating and clamp time. None of our recipients has died from a technical complication.

In conclusion, we believe that this technique could be used in humans, especially in adult uremic diabetic patients who receive a combined pancreas/kidney allotransplant from a pediatric cadaver donor.

### F117

### THE PATTERN OF PLASMA CHOLESTEROL ESTER FATTY ACIDS IS ABNORMAL IN OBSTRUCTIVE JAUNDICE

MW SCRIVEN, JCM STEWART*, DF HORROBIN*, MCA PUNTIS
Dept Surgery, University Wales College Medicine, University Wales Hospital, Cardiff, UK and *Efamol Research Institute, Kentville, Nova Scotia, Canada

We have investigated the effect of obstructive jaundice (OJ) on plasma cholesterol ester (PCE) fatty acids (FA).

PCE FA were measured in 42 patients with OJ (mean age 65, 19F: 23M, mean bilirubin 247µmol/1, 12 benign : 30 malignant) and 42 matched controls, by gas chromatography. The results were compared by Mann-Whitney U tests.

Many FA were abnormal in OJ. Overall the mean number of double bonds per FA was lower in the OJ group ($p<0.001$). This was a reflection of a fall in total polyunsaturated FA (PUFA) ($p<0.001$) and a rise in total monounsaturated FA ($p<0.001$). Total saturated FA were not different between the two groups. The change in PUFA occurred in both the major series of PUFA - n-6 PUFA ($p<0.001$) and n-3 PUFA ($p<0.002$).

This study has demonstrated marked changes in FA esterified to cholesterol in plasma in OJ. Such changes, in particular the shift towards more saturated FA, are known to impair cholesterol transport. The biological significance of these changes is unclear but they provide further evidence of the profound metabolic effects of obstructive jaundice.

### F118

### SURGICAL TREATMENT IN HYDATIC LIVER DISEASE

BERCEDO J, LOINAZ, C.JIMENEZ C, G-URENA M, PALMA F, ALVARADO A, LOPEZ A, MORENOE.

The experience of surgical treatment in hydatidosisin our service is analysed, studying the changes in our technique and results.

Since 1974-1989 410 patients with 561 cyysts were operated. We divided the period of time in two groups: GROUP A : since 1974 to 1984 (322 patients), and; GROUP B: since 1985-1989 (88 patients). We compared both groups and the total resections techniques vs partial ones. Both groups were homogeneous in age and sex.

The incisions made were: right subcostal laparotomy (A=30.7% B=43.7%); thoracophrenolaparotomy (A=25.8% B=5.7% $p<0.001$); bilateral subcostal (A=12.7%) B=32.2% $p<0.001$) supraumbilical median laparotomy (A=16.5% B=4.6% $p<0.05$) supra and infraumbilical (A=3.1% B=9.2% $p<0.05$). The surgical procedure was : hepatic resection (HR) (A=6.5% B=15% $p<0.05$); total cystopericystectomy (TQPQ) (A=64.9% B=57.5%) partial cystectomy (PQ) (A=26.3% B=27.5%); cysto-jejunostomy (A=2.1% B=0%). The total mortality was : A=2.4% B=2.1% Mortality, morbility and reoperations are shown in the Table.

The low morbi-mortality of resections techniques vs PQ is shown, so that we think total resections must be used in the hydatid liver disease (if it is possible).

| | MORTAL | FISTULA | ABCESS | REOP. | NUMBER |
|---|---|---|---|---|---|
| TQPQ | 1.15% | 3.08% | 6.15% | 3.08% | 63.4%(260) |
| PQ | 4.28% | 17.0% | 12.1% | 8.5% | 26.5%(109) |
| HR | – | 2.9% | 8.8% | – | 8.2%(34) |

$p<0.05$ between TQPQ vs QP; and between TQPQ and HR vs PQ

### V001

### REOPERATION FOR RECURRENT HEPATIC HILUM NEOPLASM

G.M. GAZZANIGA, G.BONDANZA, M.FILAURO, C.BAGAROLO, F.ERMILI
1st Department of Surgery - S.Martino Hospital, Genoa - Italy

The video shows the case of a 52 years old man, operated for klatskin tumor in an other hospital one year before. He complaints episodes of severe cholangitis, jaundice, pruritus, weight loss; pro-operative back up showed dilatation of intrahepatic biliary tree, due to obstruction of the previously confectioned hepatico-jejunostomy at the hilum. During reoperation, a tumor recurrence on the anastomosis was found, with a infiltration of externale layer of portal bifurcation. Hepatectomy was not advisable for the presence of multiple, small abscesses in the right lobe, and for small size of left lobe. Resection of intrahepatic right and left hepatic ducts, distal part of Roux-en Y-jejunal loop (with the anastomosis) was performed, in order to arrive in a free-of tumor biliary tract-resection and reconstruction of portal bifurcation was performed with an extended regional lymphadenectomy too. Biliary flow restoration was obteined with a multiple cholangiojejunostomy between second oder biliary ducts on the right and left lobe, and Roux-en Y-jejunal loop; fibercolangioscopy was performed in the post operative period (to check patency of anastomosis) using transhepatic tracks created by tranparenchimal biliary stents positioned during operations.

### V002

### EXTENDED RESECTION TO THE LEFT LOBE OF THE LIVER IN CARCINOMA OF THE HEPATIC DUCT CONFLUENCE

MORENO G.E.; CALLE S.A.; HIDALGO P.M.; SEOANE G.K.; LOINAZ S.C.; GOMEZ S.R.; VORWALD K.P.; MARCELLO F.M.
Digestive Surgery Department. Hospital "12 de Octubre". Madrid. Spain.

A patient suffering from obstructive jaundice produced by a Klatskin tumour infiltrating the left hepatic duct, was treated by means of one block resection of the common duct and left lobe of the liver extended to regional lymphonodal dissection.
The film shows the surgical steps with special emphasis to the vascular dissection of the hepato-duodenal ligament celiac axis, vena cava and abdominal aorta.

### V003

### LEFT HEPATECTOMY WITH CAUDATE LOBECTOMY FOR HILAR CHOLANGIOCELLULAR CARCINOMA

HIROSHI SHIMADA, KANJI KATAYAMA, TAIZO KOBAYASHI, GIZO NAKAGAWARA
The First Department of Surgery, Fukui Medical School, 23 Shimoaizuki, Matsuoka-cho, Yoshida-gun, Fukui, 910-11 Japan

A 59-year-old female was admitted with a complain of epigastralgia and the impairment of hepatic function. A cholangiogram by ERC and PTC showed a stricture of the left hepatic duct, and CT showed a dilation of the ascending and descending lateral hepatic duct and a low density area at he confluence of these ducts. By lateral subcostal incision was employed. The common duct was divided at the intrapancreas and the skeletonization of the hepatoduodenal ligament was done leaving portal vein and hepatic artery, dissecting regional ligament such as retropancreatic, suprapancreatic lymphnodes and intraligament lymphnodes.The left hepatic artery, left portal vein and caudate portal branches were divided. After dividing of Arantius ductus, caudate hapatic veins were divided from hilus to the root of hepatic veins exposing anterior surface of IVC. After the left hepatic vein and right hepatic duct were divided, liver splitting was done from the demarcation line of Cantlie exposing the left-side surface of middle hepatic vein to the line between posterior segment and caudate process, and the left hepatectomy with caudate lobectomy was completed. After spraying fibrin glue at the cut surface of liver, irradiation with electrone beam of 25 gray was done at the hilus including the right hepatic duct.

Hepatico-jejunostomies at superior and inferior anterior duct were done with trans-jejunal biliary drainage tube, forming Roux-Y anastomosis.

The resected specimen showed a stricture of the left hepatic duct in continuity with the mass at the confluence between ascending and descending lateral branch.

This tumour showed tubular adenocarcinoma histologically. This operation resulted in curative, and the patient is well 5 months after the operation.

### V004

### KLATSKIN TUMOUR WITH HEPATIC ARTERY RESECTION AFTER TAE

K. TAKASAKI, M. YAMAMOTO, M. TSUGITA, T. OTSUBO, T. NAKAGAMI, H. SATO, H. KOBAYASHI, H. MUTO,T. NAKAMURA, F. HANYU, S. KOBAYASHI
Institute of Gastroenterology, Tokyo Women's Medical College,Tokyo 162, Japan

Klatskin tumour, carcinoma at the bifurcation of the hepatic duct, often involves the hepatic artery. Tumour resection in these cases must therefore be supplemented by hepatic artery resection. Extension of tumour growth towards the liver parenchyma and regional vascular structures requires more extensive liver and vascular resection. Hepatic artery reconstruction in these cases is difficult. We here propose a new method to overcome these difficulties. First, TAE was performed, followed 2-3 weeks later by resection. No hepatic artery reconstruction was done. Three cases underwent this schedule, with satisfactory postoperative follow-up. Depending on the extent of neoplastic infiltration, tumour resection is sometimes combined with liver resection and pancreatoduodenectomy. This time we present a case in which this extended operation was performed.

### V005

### LIVER TRANSPLANTATION WITH PRESERVATION OF RECIPIENT IVC IN A PATIENT WITH A PORTOCAVAL SHUNT

C. MARGARIT, J. BALSELLS, R. CHARCO, JL. LAZARO, E, MURIO, I. DIAZ, A. EDO, J. BONNIN-SANCHEZ.
Liver Transplantation Unit. Department of Surgery, Hospital General Vall d'Hebron. Universidad Autonoma. Barcelona, Spain

We present a video of the technique of liver transplantation with preservation of the recipient inferior vena cava in a 55 years old female patient diagnosed of postnecrotic liver cirrhosis who underwent a portocaval shunt one year before. Recipient hepatectomy is performed maintaining the portocaval shunt in place and dissecting the inferior vena cava from the liver. A vascular clamp is placed at the level of the hepatic veins. During the anhepatic phase there is no hemodynamic compromise because both the porta and caval flow are kept, therefore no venovenous bypass is needed. Revascularization of the new liver begins with the suprahepatic IVC anastomosis, then portocaval shunt is taken down and the portal anastomosis is fashioned. The infrahepatic IVE is sutured. The arterial and biliary anastomoses are done in the usual way. This technique was developed for the segmental liver transplantation in children. It is an ideal technique for patients with a portocaval shunt who cannot tolerate the clamping of the portal vein during longtime and where a venovenous bypass was used routinely before.

### V006

### SYNCHRONOUS LIVER & KIDNEY TRANSPLANTATION

MORENO G.E.; GARCIA G.I.; GOMEZ S.R,; G. PINTO A.I.; LOINAZ S.C.; JIMENEZ R.C.; IBAÑEZ A.J.; BERCEDO M.J.; PEREZ-CERDA F.; RIAÑO C.D.
Digestive Surgery Department. Hospital "12 de Octubre". Madrid, Spain

A patient suffering from kidney chronic insufficiency was treated by kidney transplantation. Hyperacute rejection without response to medical treatment was finally avoided by means of resection of the graft going back to haemodialysis during seven years producing postnecrotic cirrhosis, with untreatable ascites and bleeding oesophageal varices.

The film shows the operation designed: synchronous liver & kidney transplantation. First, liver resection was done and a total liver graft was anastomosed to the IVC, portal vein and biliary tract when the liver transplantation finished, a kidney from the same donor was implanted in the left iliac fossa.

The film shows the postoperative test and the aspect of the patient four years after the operation.

### V007

### LIVER TRANSPLANTATION WITHOUT RESECTION OF THE RECIPIENT VENA CAVA

E. VICENTE, V.S. TURRION, J. NUÑO, F. PEREIRA, J. HERRERA, N. PEREZ, J. VAZQUEZ, J. ARDAIZ
Interhospital Liver Transplant Unit. Clinica Puerta de Hierro, Hospital Infantil La Paz, Hospital Ramón Y Cajal. Madrid, Spain

Liver transplantation is the treatment of choice for end-stage liver disease.

The surgical technique of orthotopic whole liver transplantation is well known. The liver is removed with interruption of portal vein, inferior and superior vena cava. The alterations derived from the vascular clampage has been avoided by using an active veno-venous bypass without heparin.

Technical progress has been observed in the last years. In this sense, one of the most effective alternatives to standard procedure is the preservation of the recipient vena cava. Mainteinance of blood flow through the vena cava during the anhepatic phase will allow the majority of consequences of the above mentioned clampage, as well as obviating the routine use of a veno-venous by-pass.

The video shows the orthotopic liver transplantation with preservation of the recipient vena cava carried out on a 57 year-old patient, affected by a post-necrotic liver cirrhosis.

### V008

### IS SEVERE OBSTRUCTIVE CHOLECYSTITIS WITH EMPYEMA A CONTRAINDICATION TO LAPAROSCOPIC CHOLECYSTECTOMY (LC) ?

H.J. ESPINER, W.K. ELTRINGHAM. A. ROE. R. MILLER
Bristol Royal Infirmary, England.

When obstructive cholecystitis is complicated by empyema or fistula standard dissection in Calot's triangle may be impossible; for this reason most authors continue to exclude such cases from LC. We have devised a method which has allowed the operation to proceed safely in 17 cases. Omental adhesions are separated by gentle blunt dissection to expose Hartmann's pouch. Dissection is continued on the left side of the pouch using discreet spot diathermy to seal small vessels directly on the wall. If a narrow cystic duct is not immediately visible beneath the impacted stone central dissection is abandoned. Instead an incision is made directly onto the stone from the freed outer surface, releasing it and the gall bladder contents: large stones are placed in a retrieval sac deployed on the anterior surface of the liver. The incision then encircles Hartmann's pouch transecting it safely from within its lumen; a dissection plane in the liver bed is then easily identified. Central dissection is fully under control and can proceed according to the anatomy: ligation of the cuff or simple drainage are safe options. This technique avoids opening a duodenal fistula or damaging an adherent duct and offers a possible solution for the Mirizzi syndrome.

Results: There have been no significant complications. Two cases had common duct stones: one was treated by direct CBD extraction during LC, and the other by ERCP postoperatively.

It is concluded that complicated obstructive cholecystitis is not an absolute contraindication to LC.

### V009

### INTRA OPERATIVE ERCP FOR CHOLEDOCHOLITHIASIS DURING LAPAROSCOPIC CHOLECYSTECTOMY.

M. GAGNER, E. DESLANDRES, A. POMP

Dept of Surgery and Gastroenterology, Hôtel-Dieu de Montréal, Univ. Montreal

Laparoscopic trans-cystic is performed for choledocholithiasis found on routine cholangiography during laparoscopic cholecystectomy. This approach cannot be performed if the cystic duct is not dilated or dilatable, if the cystic duct insertion is too low or if choledocholithiasis are located in the common hepatic duct or the upper biliary tree. ERCP is often performed postoperatively when the trans-cystic exploration fails. This study was performed to assess the feasibility and success of intraoperative ERCP during laparoscopic cholecystectomy. Ten patients had choledocholithiasis during routine cholangiography and underwent ERCP with sphincterotomy. A dormia basket catheter was inserted in the common bile duct for stone extraction, and cleared the duct in all patients. The ampulla was located with the cholangiography catheter in 9 patients and helped perform the sphincterotomy. There were no deaths and no major complications. Two patients had transient hyperamylasaemia. The median postoperative stay was 3 days (range 2-7 days) and the median operative time of combined procedures was 100 minutes range (80-100 minutes). Seven patients had a single stone (average size of 10 mm) and 3 patients had multiple stones. These preliminary results on intraoperative ERCP during laparoscopic cholecystectomy confirm the feasibility and success of this technique for the treatment of associated choledocholithiasis.

### V010

### APPLICATION OF ULTRASONIC ASPIRATOR FOR LAPAROSCOPIC CHOLECYSTECTOMY

N. KANO, T. YAMAKAWA, S. SAKAI, Y. ISHIKAWA, H. HONDA,S. OHTAKI, A. TACHIBANA, A. OHMURA*, M. SHA*
Department of Surgery and Department of Anesthesiology*,Teikyo University Hospital at Mizonokuchi, Kawasaki, Japan

Ultrasonic aspirator combining multiple functions including irrigation, suction, cutting and coagulation have been developed with a close cooperation of the Olympus Optical Co. to use for laparoscopic surgery. In this paper, our experience with laparoscopic cholecystectomy using these instruments is presented in video.

The hand piece, 10mm in diameter, is air tight and can be inserted via 10mm trocar. It installs ultrasonic probe, 2.3mm in diameter, with capacity of 23.5 KHz vibration frequency and 300 pm amplitude in maximum controlled by foot switch. A capability of suction and irrigation using saline are operated by foot switches. Moreover it is connectable to the high frequency generator and either cutting of tissue or coagulation of bleeder can be easily manipulated by hand switches without an exchange of hand piece.

Forty to 60% of maximum vibration frequency is sufficed for laparoscopic cholecystectomy. Tissue selectivity of ultrasonic aspirator preserving small vessels makes it easier to explore the Carot's triangle and skeletonize both the cystic artery and duct.

The use of this instrument with multiple functions which can be manipulated without an exchange of the handpiece makes laparoscopic cholecystectomy safer and easier. Therefore, it is considered to be very useful and effective to use this instrument to prevent major complications in laparoscopic cholecystectomy, because it can be precisely carried out with less bleeding and head injury of tissue.

### V011

### LAPAROSCOPIC SURGERY FOR POLYCYSTIC LIVER DISEASE

G. GOZZETTI, A. MAZZIOTTI, A. PRINCIPE, E. JOVINE, M. MORGANTI, G.L. GRAZI
Clinica Chirurgica 2°, University of Bologna, Bologna, Italy

Polycystic liver disease can occasionally be an indication for surgery, especially when pain or symptoms by compression occurred. These symptoms are usually related with the presence of large cysts deforming the profile of the liver. Conventional surgery includes the so-called "fenestration", that is the opening of the cystic wall and the removal of its exuberant portion. Nowadays this procedure can be performed through a laparoscopic way. The video shows the case of a 45 year-old lady carrying polycystic liver disease; she complained severe symptoms for the presence of a large number of cysts appearing at the liver surface and arriving to the pelvic cavity. Under laparoscopic guidance the cysts were incised, emptied and the external wall was removed by the scissor and the dissecting hook. The post-operative course was uneventful, without the need of any post-operative analgesic. The patient was eventually discharged on the fourth postoperative. day.

(Video; 12 minutes)

### V012

### LAPAROSCOPIC COMMON BILE DUCT EXPLORATION

SUNG GYU LEE, MYUNG-HWAN KIM, YOUNG-IL MIN, KU-BO SUNG, PYUNG-CHUL MIN
Gallstone study Group, Asan Medical Center, Ulsan Medical School, Songpa-kus Poongnap-dong 388-1,138-040, Seoul, Korea

500 laparoscopic cholecystectomies have been performed to treat the gallstone disease at the Asan Medical center between September 1990 and December 1991.

Initially, laparoscopic cholecystectomy has been tried for the treatment of uncomplicated symptomatic gallstone disease, but now we have carefully widened the indication of laparoscopic cholecystectomy and tried for the management of common bile duct stones, and intrahepatic stones.

Laparoscopic management of the common bile duct stones was attempted and accomplished successfully in 19 patients by 18 laparoscopic common duct exploration and 1 fluoroscopic stone removal through the cystic duct.

The stones could be removed either by a nelaton catheter irrigation, a #6 balloon Fogarth angiocatheter, of a 4.8mm flexible choledochoscope using Dormia basket.

The postoperative course of these patients was exactly same as those simple laparoscopic cholecystectomy except the discomfort coming from the T-tube insertion for 2 weeks.

Laparoscopic common bile duct exploration is an effective and safe procedure for the management of choledocholithiasis seems to be preoperatively so they can be extracted by endoscopic sphincterotomy.

### V013

### SPLENIC PRESERVATION IN THE MANAGEMENT OF CONGENITAL CYSTS

S.S HANNA
Division of General Surgery, Surgery, Department of Surgery, Sunnybrook Health Science Centre, University of Toronto, Toronto, Ontario, Canada

Non-parasitic, non-neoplastic, congenital splenic cysts are rare. It is therefore likely that abdominal surgeons dealing with adult patients will encounter such patients rarely, if ever, during their professional careers. The French surgeon Jules Peau performed the first splenectomy for a splenic cyst in 1867. By 1952 Fowler was able to collect 265 cases of splenic cysts from the literature. Since then isolated reports and reviews have brought the total to over 600. Some surgeons are still performing total splenctomy for isolated splenic cysts. Partial splenectomy is recommended since it preserves the important immune functions of the spleen.

The enclosed video demonstrates the procedure of upper pole partial splenectomy with preservation of the lower pole of the spleen in a 21-year old female. She underwent an unsuccessful marsupialization of her splenic cyst at age 14. She complained of left upper quadrant abdominal pain and early satiety due to gastric compression. We utilized the CUSA-Cavitron in the splenic transection and found it useful in minimizing blood loss. Total blood loss was about 300 cc. The patient is now 23 months postop. and continues to be asymptomatic with normal postoperative ultrasounds showing no residual or recurrent splenic cysts.

### V014

### COMPLETE DENERVATION OF THE PANCREAS FOR CHRONIC PANCREATITIS WITHOUT PANCREATIC DUCT DILATION

T. HIRAOKA, K. KANEMITSU I. KAMIMOTO, Y. MIYAUCHI
First Department of Surgery, Kumamoto University Medical School, Kumamoto, Japan

We studied pathophysiologic findings of chronic pancreatitis without dilatation of the main pancreatic duct. Patients with such disease were divided into two groups, that is, segmental lesion group and difuse lesion group. Patients with the difused lesion had far advanced dysfunction of the pancreas, but some cases of them had severe pain. Therefore, with the aim of pain control in such cases without the morbidity of insulin-dependent diabetes, a new procedure was devised to completely resect the postganglionic pancreatic nerves and to totally free the pancreas from the posterior abdominal wall. This procedure was performed on three patients with follow-up periods of more than 2 years. Pain was resolved in all the patients, and their blood glucose levels were substantially unchanged. This new approach offers a means of relieving pain with preservation of endocrine function in selected patients with chronic pancreatitis, especially in patients who have a small pancreatic duct. We would like to introduce in detail of this procedure.

### V015

### WHIPPLE OPERATION WITH PANCREATICO GASTRIC ANASTOMOSIS: A SAFE METHOD OF RECONSTRUCTION

CD JOHNSON
University Surgical Unit, Southampton General Hospital, Southampton UK

The pancreatic anastomosis after Whipple resection is the source of much morbidity and is a major cause of mortality. Review of the recent literature shows a 14% fisutla rate and 7% mortality with this reconstruction. One third of all deaths were caused by pancreatic fisutula. In contrast the reported rate of leakage from pancreaticogastric anastomosis is much lower (1 of 128 cases)

This video describes the technique of pancreatic head resection with end to side pancreaticogastric anastomosis.

In 50 cases, performed for pancreatic adenocarcinoma (21), ampullary adenocarcinoma (13), bile duct tumours (10), and other conditions (6) there were 2 deaths (haemorrhage 1, sepsis1). There were two pancreatic fistulas which closed after 5 days and two weeks.

### V016

### CYSTIC TUMORS OF THE PANCREAS

D.MARRANO
Instituto di 1^ Clinical Chirurgica Generale e Terapia Chirurgica - Universita degli Studi di Bologna
Policlinico S. Orsola - Via Massarenti, 9 - 40138 Bologna (Italy)

Cystic Tumors of the Pancreas (CTP) represent an interesting chapter of surgical pathology for recent histological, clinic, prognostic and therapeutic acquisitions. From 1982 through 1991, 22 cases of CTP were observed at 1^ Clinica Chirurgica, Bologna University, representing 18.6% of 118 cystic lesion of the pancreas and 7.9% of 277 cases of pancreatic exocrine tumors. One serous microcystic tumor, 5 mucinous cystoadenomas, 13 mucinous cystoadenocarcinoma, one solid and cystic papillary tumor and 2 intraductal papillary neoplasms were observed. Radical operation was carried out in 19 cases (86.4%) While palliation and laparotomy was performed in 2 and 1 case respectively. Radical operations consisted in 11 left pancreatectomy, 2 sub-total pancreatectomy, 4 pancreaticoduodenectomy, and 2 total pancreatectomy. Cumulative survival rate was 78.6% at 2 years and 62.5% at 5 years. As a result of these data we can conclude that: a) CTP are not rare tumors; b) new knowledges about histological pattern are emerging; c) pre - and intraoperative diagnosis can precisely define the exact nature of the cystic lesion; d) surgery must be carried out as a radical operation for the high resectional index; e) prognosis of CTP is good comparing to the other tumors of the pancreas.

### V017

### TRANSDUODENAL WIRSUNG SPHINCTEROTOMY + LONGITUDINAL SIDE-TO-SIDE PANCREATICOJEJUNOSTOMY

D. MOLINO,V. NAPOLI,G. PATERNITI,M. MARESCA,S. MOSCA
Dep. of Surgery and Pancreas Surgery Unit, Ospedale Cardarelli, - Napoli, - Italy

A 62-old patient operated 3 years before of pancreatic cyst removal (we are not sure of this) and suffering from chronic pancreatitis has been undergone to this double procedure: 1) longitudinal pancreaticojejunostomy utilizing a defunctionalized segment of jejunum being the pancreatic duct over 1 cm.

2) transduodenal Wirsung-sphincterotomy.

Even if Wirsung sphincterotomy have little application in the treatment of chronic pancreatitis, it has been employed in this case because of the stricture and fibrosis of the duct in the first portion of pancreas head. We think that the association of these two surgical procedures can be the best solution for the treatment of chronic pancreatitis and pain relief.

### V018

### A NEW METHOD OF PYLORUS-PRESERVING PANCREATODUODENECTOMY

G. DE BERNARDINIS, A. AGNIFILI, P. GOLA, I. IBI, R.VERZARO, S. GUADAGNI, F. GIANFELICE, R. FANINI
Department of Surgery*, Department of Oncology, University of L'Aquila, Italy

Acute pancreatitis and leakage of pancreatic-jejunal anastomosis are the most important causes of operative morbidity and mortality after pancreatoduodenectomy.

We have introduced a modified technique for the reconstruction that provides the functional exclusion of the pancreaticjejunostomy, respect to the transit of gastric and biliary secretions. The pancreatic-jejunostomy is made on a separate loop respect to the duodeno-jejunal and hepatic-jejunal anastomoses. The immediate advantages are the reduction in risk of leakage and the possibility of its conservative treatment, in case leakage occurred.

Preservation of antral-pyloric unit, according to Traverso-Longmire, increases the functional features of the procedure, by reducing entero-gastric refluxes, and assuring a regulated gastric emptying.

We present our series of 11 pancreatoduodenectomy(PD) for periampullary neoplasms and chronic pancreatitis. We didn't have any operative mortality.

The specific morbidity consists of one case of external biliary fistula by micro-dehiscence of the hepatic-jejunostomy resolved conservatively. Late results confirm almost normal findings in terms of gastric secretion, gastric emptying and absence of dumping syndrome, ulcers and refluxes.

References

Machado M.C.C., Monteiro da Cunha J.E., Bacchella T., Bove P. and Raia A. A modified technique for the reconstruction of the alimentary tract after pancreatoduodenectomy.

Surg. Gynecol. Obstet. 1976;143:271-272.

### V019

### RADICAL DUODENOPANCREATECTOMY PLUS REGIONAL LYMPHONODAL DISSECTION

MORENO G.E.; GOMEZ S.R., GARCIA G.I., LOINAZ S.C., GONZALEZ-PINTO A.I.; JIMENEZ R.C.; BERCEDO M.J.; PALMA C.F.; PALOMO S.J.C.; HERNANDEZ G.D.
Digestive Surgery Department. Hospital "12 de Octubre".
Madrid, Spain.

The film shows the different steps of regional duodenopancreatectomy in pancreas carcinoma. Special remarks are shown in the retroperitoneal dissection for one-block resection.

Reconstruction was done by means of hepaticojejunostomy (end-to-side) and gastro-jejunostomy.

### V020

### PANCREATICODUODENECTOMY WITH THE INTRAOPERATIVE RADIATION THERAPY FOR PANCREATIC CANCER

K. SATO, M. YOSHIDA, K. FURUTA, T. SUZUKI, H. IZUMIKA, H. YOKOTA, S. FUNAMOTO, G. KANEDA, K. ASO, H. OHMIYA, Y. HIKI, A. KAKITA
Department of Surgery, Kitasato University, School of Medicine, Kanagawa, Japan.

Recently the accuracy of the diagnosis and resection rate in the pancreas cancer have been developed by the development of the diagnostic imaging and the advanced surgery. However 5-year survival rate is still low. Diagnostic imaging and autopsy showed high frequency of local recurrence and liver metastasis after surgery of pancreatic cancer. Intraoperative radiation therapy might be useful to prevent local recurrence.

This case was 48 year-old male. He was admitted in the condition of obstructive jaundice. Total bilirubin was 3.5 mg/dl with direct bilirubin being 2.6 mg/dl. The pancreatic head tumour was found with echography, CT scan and other diagnostic imaging.

Pancreaticoduodenectomy was performed combined with intraoperative radiation. Radiation therapy was performed right after the resection of pancreaticoduodenal tissues. After radiation, reconstractive surgery was performed with Child procedures. In conclusion intraoperative radiation might be useful to prevent local recurrence of pancreatic cancer. Improving of survival rate of pancreatic cancer is expected with intraoperative radiation therapy.

### V021

### SEGMENT III LEFT DUCT ANASTOMOSIS FOR BILIARY-ENTERIC DRAINAGE IN NEOPLASTIC HIGH BILE DUCT OBSTRUCTIONS

DE SOUZA L.J., JAGANNATH P, SHANTI SWAROOP V,
Gastrointestinal Service, Tata Memorial Hospital, Parel, Bombay-400 012, India

High bile duct obstructions are most often locally advanced when detected and are not usually amenable for resection. Very effective drainage can be obtained by using the segment III left duct for biliary enteric anastomosis.

A transverse subcostal incision provides good exposure. If the lesion is unresectable and the common hepatic duct cannot be identified, the segment III left duct is exposed. This is made easier by lowering the hilar plate. The ligamentum teres is pulled down and the fissure is opened. The segment III left duct is then seen running across the fissure at the lower end and is relatively extra-hepatic. The duct is confirmed by aspiration. A one cm longitudinal opening in the duct is made. A Roux-en-Y loop of jejunum is prepared of at least 50 cms and an end to side biliary enteric is made, using one layer of 3-0 interrupted silk sutures. A No. 8 plastic Ryle's tube stent is kept across the anastomosis and brought out through the jejunal loop. It is usually removed after 10 days.

In our experience, this technique provides effective biliary drainage and affords good palliation even in advanced cases of neoplastic high bile duct obstructions.

### V022

### A SIMPLIFIED TECHNIQUE FOR INTRAOPERATIVE CHOLANGIOGRAPHY IN LAPAROSCOPIC CHOLECYSTECTOMY

SUMIO INOUE, YUICHI ISHIDA, TAKESHI NAGAO, HISANORI UCHIDA
Institute of Medical Science, Tokyo, Japan

Laparoscopic cholecystectomy (LC) is being so favourably accepted as if to replace ordinary "open" cholecystectomy before long. However, LC has been reported to be associated with some potential risks, of which injuries to the bile duct would be the most serious. Although performing intraoperative cholangiography has been advised prior to transecting the "presumed" cystic duct (CD), cannulating CD may need fully skilled hand and yet could be hazardous should the duct be the common bile duct. Thus, we developed a new method of cholangiography for LC. The device is consisted of two parts: a guide sheath that fits into 5-mm SURGIPORT™ and a soft tube tipped with a 1 cm length fine needle. The outer end of the sheath is so devised as to keep the intraperitoneal gas from escaping and to permit a smooth move of the tube within the sheath as well. Dissected CD is punctured with the needle connected to the tube through which contrast material is injected. Even if the punctured duct is not CD, such small injury is presumed to be safely left untreated. The first attempt with this device was successfully made on the author's second case with LC. In summary, our puncture method, unlike a popular cannulation method, can warrant quick and safe intraoperative cholangiography and is expected to help achieve safer laparoscopic cholecystectomy.

### V023

### DEFINITIVE TREATMENT OF IATROGENIC LESIONS OF THE COMMON DUCT PREVIOUSLY TREATED

MORENO G.E.; RICO S.P.; GOMEZ S.R.; GARCIA G.I.; LOINAZ S.C.; JIMENEZ R.C.; GONZALEZ-PINTO A.I.; BERCEDO M.J.; IBANEZ A.J.; ALVARADO A.; VEGA R.V.; GARCIA U.M.A.; MORENO S.C.
Digestive Surgery Department. Hospital "12 de Octubre". Madrid, Spain.

Two patients with surgical lesions of the biliary tract previously treated by different techniques without success are included in this film.

The previous operations are analysed and the definitive operation purpose is discussed.

Dissection of the biliary tract, mobilization of a Roux-en-Y jejunal loop, resection of the common duct and dissection of the main two branches are shown.

A very large opening in both hepatic ducts was obtained. The side-to-side cholangio-jejunostomy was performed.

Special emphasis in the different reconstructive steps was done.

### V024

### THE SURGICAL TREATMENT OF BILIARY CYSTS

G. GOZZETTI, A. PRINCIPE, A. MAZZIOTTI, E. JOVINE, M.L. LUGARESI, F. RUBERTO
Clinica Chirurgica 2°, University of Bologna, Bologna, Italy

Biliary cysts are a rare congenital disease occurring in all the biliary tree. The cysts are classified according to the Todani's method. The high occurrence of carcinoma leads to the complete surgical excision when feasible. The video shows three operations carried out for each type of malformation observed: 1 respectively for Type I , IVa and V. The importance of intraoperative ultrasound and cholangiography is stressed to guide the surgical strategy in the case of multiple intrahepatic localizations. In type I cyst the complete excision was performed and the biliary tree reconstructed with a Roux-en-Y hepaticojejunostomy. In the Type IVa cyst, with multiple localizations into the posterior liver segments, the resection of the extrahepatic cyst and a biliary digestive anastomosis at the hilum were carried out. In type V cyst, thanks to intraoperative ultrasonography, the initially planned left lobectomy was extended and a left hepatectomy performed. The video also presents the good results in the complete series of 15 patients (12 female and 3 male).

(Video, 15 minutes)

### V025

### BUDD-CHIARI SYNDROME – PATHOGENESIS AND TREATMENT

K.H. ZHANG, Z.Y. GU
Department of Surgery, Chinese PLA General Hospital, Beijing, China

Abstract: Of 68 patients with Budd-Chiari syndrome treated by us, 66 cases had changes of inferior vena cava (IVC), which we divided into three categories of obstruction by cavography, ie, stenosis of hepatic IVC (Type I); occlusion of IVC at hilum of diaphragm (Type II); and obliteration of hepatic IVC (Type III). Research of autopsy in six cases showed that congestion of liver and sequential cirrhosis was the major changes to be solved. 50 cases were operated upon with three kinds of operation: 1) portosystemic shunt was for two patients with type I; 2) cavoatrial, azygoatrial and mesoatrial bypass were for type II in 19, 1, and 1, respectively; 3) splenopneumopexy was elected for 18 patients with type III, and remainings were with other procedures. There were not operation death and seldom complications. Follow-up study showed that a shorter graft for by-pass led a better result. Pathogenesis of such entity and option to be elected for treatment were also discussed.

(Video, 14 minutes)

**V026**

### MAS IN THE MANAGEMENT OF GALLBLADDER AND MAIN DUCT STONES

E. CROCE, I.NOVELLINO, M. AZZOLA, M. LONGONI, G.PALAZZINI
Ospedate Fatebeneitrateli, I Divisione Chir, Milano - Italy
III Clinica Chirurgica - University "La Sapiezza" -Rome, Italy

In the videotape our experience in the management of gallbladder and main duct stones with MAS is shown.

Although different therapeutical options are available, currently our choice is for a sequential treatment that is preliminary endoscopic papillotomy for main duct clearance followed, in a short time, by laparoscopic cholecystectomy.

For such treatment we must preoperatively know if a main duct lithiasis is present, therefore, we don't perform routine,i.e. cholangiography but patients undergo preoperative cholangiography and/or ERCP in case of doubts.

Up to October 1991, 30 patients were treated using this method. Even though several of them were at high risk no mortality and a very low morbidity were registered, with the further advantage of a short hospitalization period.

Other therapeutical options including transcystic exploration with Nefaton and Forgarty catheters under X-ray control and choledocoscopy are shown.

Indeed, in our opinion, the ideal treatment, as shown, involves a complete exploration of the biliary system with stone extraction and primary suture of the main duct at the time of laparoscopic cholecystectomy. As soon as this method will further demonstrate its accuracy we believe that, at least in well experienced centers, it will represent the ideal procedure.

**V027**

### CT-FLUOROSCOPY (CTF) IN THE DIAGNOSTIC AND THERAPEUTIC APPROACH TO HEPATIC LESIONS

L.F FRIGERIO, C. SPREAFIGO, A. MARCHIANO', M. MILELLA, F. ZUCCHI AND B. DAMASCELLI
Special Radiologic Procedures Dept. - Istituto Nazionale Tumori - 1, v. G.Venezian - 20133 Milano - Italy

We have coupled a digital subtraction image intensifier to a CT scanner. The patient is lying on the CT table and an usual Seldinger maneuver is performed for positioning the angiographic catheter into the superior mesenteric artery. Then a CT arterial portography is performed so that the liver is opacified from the portal system. After that a complete angiographic study is carried out; the X-ray unit is provided with a tape recorder and a multiformat camera for the documentation. If necessary, a pre-surgical or definitive chemoembolization can be carried out in the same session and CT provides an immediate control of the path of the emboli.

This method has shown some advantages: a) it allows the real-time fluoroscopic control of the angiographic maneuvers, b) it reduces the time requested for the two studies, c) the entire procedure can be carried out in one room without moving the patient; d) it allows a smoother handling of patients at risk.

**V028**

### SURGERY FOR HYDATID LIVER CYSTS

G. GOZZETTI, A. MAZZIOTTI, E. JOVINE, G.L. GRAZI, A. FRENA, A. CAVALLARI
Clinica Chirurgica 2°, University of Bologna, Bologna, Italy

This video shows a total pericystectomy for a parasitical cyst located in the lateral segment of the liver and a subtotal pericystectomy for a huge cyst occupying the medial segments. The importance of intraoperative ultrasonography is stressed in recognize the relationships between the pericyst and the intra-hepatic vascular anatomy. The video also presents the results of different techniques employed in 120 patients treated over the past 10 years. The importance of a more radical approach, living in place as less pericystic wall as possible, has a direct influence on the post-surgical morbidity and on hospital stay.

(Video, 14 minutes)

**V029**

### RADICAL SURGERY IN THE TREATMENT OF HEPATIC HYDATIDOSIS

E. VICENTE, M. DEVESA, J. NUÑO, V. ANGEL, F. PINIELLA, F. GALBIS, V. GARRIDO

Hospital Ramón y Cajal, Madrid, Spain

Several surgical techniques have been used in recent years for treatment of hepatic hydatidosis of the liver, that surgeon select guided by the number, size, localization of cysts as well as the experience in liver surgery.

The complete removal of wall and cyst contents is the choice technique in the surgical treatment.

Liver surgery has evolved rapidly in the past 2 decades. The increased safety of hepatic resections has resulted from a number Of improvements, including technological changes.

The video shows the total pericystectomy operation carried out on two patients affected by hepatic hydatid cysts with partial vascular control and the use of the ultrasonic surgical aspirator in the hepatic parenchymal division.

### V030

### IMPROVEMENT OF SURGICAL TREATMENT OF HYDATID DISEASE OF THE LIVER

XU MING-QIAN
Research unit of hydatidology of people's hospital of Xinjiang Autonomous Region, Urumqi, 830001 P.R. China

There were 1,640 patients with hydatid disease who underwent surgical treatment in our hospital during 1953-1990. of these, 1,204 cases were liver hydatid disease, and 1,319 operations yielded 1,716 Echinococcus cysts. The methods of removal of hydatid cyst were: 1) hepatic lobectomy 2) removal of intact hydatid cyst 3) removal of hydatid cyst by aspiration.

Removal of the cysts left in the liver residual ectocyst cavities as large as the cysts and bile-containing bloody retention cyst, usually resulted in secondary infection, causing liver abscess. The clinical observations and experimental studies of this series revealed the mechanism of intrahepatic biliary fistula formation and the natural rule of cavity closure, allowing rational cavity management and modification of operative methods. This reduced the frequency of postoperative biliary fistula and of secondary infection, and also shortened the healing period.

### V031

### HYDATID DISEASE OF THE LIVER: CISTOPERICISTECTOMY

PROF. V. ROVATI, DR, E. FALESCHINI, DR. G. NERVETTI, DR. G. TAGLIABUE, DR. F, COLTURANI.
Istituto Scienze Biomediche L. Sacco - Catt. Patologia Chirurgica - Universita di Milano - Italia

The video shows a case of hydatidosis of the left hepatic lobe, appearing on the surface of the parenchyma. Cisto-pericistectomy was performed. This is the treatment of choice, because it doesn't cause reduction of the functional parenchyma and has better possibilities for radical.

### P001

### NEONATAL BILE DUCT PERFORATION AND STRICTURE: TWO OF A KIND?

W.A. BEMELMAN, H.A. HEY, A. VOS and M.N. van der HEYDE
Departments of Paediatric Surgery and Surgery, Academic Medical Center, University of Amsterdam, 1105 AZ Amsterdam

Benign bile duct strictures are occasionally seen in early infancy. Usually, no cause can be identified and the stricture is said to be "congenital". Spontaneous bile duct perforation is a rare entity which occurs in the first 12 weeks after birth. This disease may present as a subacute illness with jaundice, acholic stools, failure to thrive and progressive biliary ascites. A pseudocyst may be found in the hepatoduodenal ligament at laparotomy. Both spontaneous perforation and the stricture of the duct tend to be located just proximal of the junction of common bile duct and cystic duct. We have analyzed our patient material in order to asses whether infants treated for a neonatal bile stricture could have had a bile duct perforation prior to the clinical manifestation of the stricture. Three cases were found. Two of them had a Roux-en-Y hepaticojejunostomy for a bile duct stricture located at the junction of the common bile duct and the cystic duct at an age of three and 20 months respectively. One infant had a transient period of moderate jaundice associated with dilated intrahepatic ducts at an age of one week. The other child had a period of melaena and haematemesis presumably caused by haemobilia two months after birth. In both cases the symptoms resolved spontaneously. The third infant had an explorative laparotomy for extrahepatic cholestasis five weeks after birth. A pseudocyst filled with bile was found which was located in the hepatoduodenal ligament. The cyst was drained. The perforation site could not be identified. Two weeks after surgery, a biliary fluid collection was drained percutaneously. These cases suggest that spontaneous bile duct perforation might pass unobserved when located in the hepatoduodenal ligament, and might proceed to a bile duct stricture.

### P002

### RESULTS OF RECONSTRUCTIVE PROCEDURES FOR BILE DUCT INJURIES AND BENIGN STRUCTURE

COLOVIC R., SAVIC M., MILICEVIC M. AND BILANOVIC D.
Institute for Digestive Diseases, University Clinical Center, Belgrade 11000, Yugoslavia, Koste Todorovica br. 6.

The aim of this retrospective study is to evaluate the long-term results of reconstructive procedures for bile duct injuries and benign strictures. A total of 86 pts. were analyzed, 57 women (66.3%) and 29 men (33.7%). Two pts. had congenital bile duct stricture, 6 had stricture due to chronic pancreatitis and 78 pts. had bile duct injuries. One bile duct injury was due to an abdominal penetrating wound and 77 were iatrogenic (12 "acute" injuries and 65 presenting as postoperative benign strictures). In 66 pts. bile duct injury occurred during cholecystectomy, in 9 during gastrectomy and in 2 during operation for liver hydatid disease. Two pts. had disinsertion of the papilla of Vateri and 75 pts. had section or resection of the bile duct. Unsuccessful previous reconstructive procedures were done in 52 pts. 1 to 6 times. According to the Bismuth classification 25 pts. (29.0%) were type I, 25 pts. (29.0%) were type II, 23 pts. (26.9%) were type III, 12 pts. (14.0%) were type IV and 1 pt. (1.16%) was type V. Six pts. had intrahepatic bile duct stricture and 25 pts. had intrahepatic stones. Reconstruction of the previously done Roux-en-Y jejunal limb was necessary, since it was found to be too short, in the majority of pts.

The reconstructive procedures in the 12 pts. with "acute" injuries were: 1 implantation of the papilla into the duodenal stump and 1 into a Roux-en-Y loop, 8 end-to-end anastomosis over a "T" tube brought out through a separate bile duct incision. Reconstructive procedures in the 74 pts. with postoperative benign strictures were: 75 cm long Roux-en-Y hepaticojejunostomy in 67 pts., choledochoduodenostomy in 3 pts., choledochoplasty in 1 pt. and resection of stricture with end-to-end anastomosis in 2 pts. Reconstruction was impossible in 1 pt.

Overall operative mortality was 1.2%. The pt. with portal vein thrombosis in whom reconstruction was technically impossible, died 4 months after operation due to uncontrollable variceal bleeding. Two pts. died three years after successful reconstruction due to unrelated causes. All remaining pts. are alive and entered into a follow-up protocol. The average follow-up period was 5 years (median 4.2 years). According to Blumgart's criteria good results were achieved in 76 pts. (89.4%), satisfactory in 6 pts. (7.0%) and unsatisfactory results in 3 pts. (3.5%). One pt. with unsatisfactory results was reoperated and later entered into the "good results" group. Factors influencing outcome were the level of stricture, portal hypertension, time from injury to definitive operation, number of unsuccessful previous attempts at reconstruction and the hypertrophy/atrophy complex. The authors conclude that the majority of "acute" bile duct lesions and benign bile duct strictures can be managed successfully.

